# Mislocalization of centromeric histone H3 variant CENP-A contributes to chromosomal instability (CIN) in human cells

**DOI:** 10.18632/oncotarget.18108

**Published:** 2017-05-23

**Authors:** Roshan L. Shrestha, Grace S. Ahn, Mae I. Staples, Kizhakke M. Sathyan, Tatiana S. Karpova, Daniel R. Foltz, Munira A. Basrai

**Affiliations:** ^1^ Genetics Branch, CCR, NCI, NIH, Bethesda, MD, USA; ^2^ Biochemistry and Molecular Genetics, University of Virginia, Charlottesville, VA, USA; ^3^ Laboratory of Receptor Biology and Gene Expression, CCR, NCI, NIH, Bethesda, MD, USA; ^4^ Department of Biochemistry and Molecular Genetics, Northwestern University, Chicago, IL, USA

**Keywords:** chromosomal instability, centromeres, cancer, CENP-A, DAXX, Chromosome Section

## Abstract

Chromosomal instability (CIN) is a hallmark of many cancers and a major contributor to tumorigenesis. Centromere and kinetochore associated proteins such as the evolutionarily conserved centromeric histone H3 variant CENP-A, associate with centromeric DNA for centromere function and chromosomal stability. Stringent regulation of cellular CENP-A levels prevents its mislocalization in yeast and flies to maintain genome stability. CENP-A overexpression and mislocalization are observed in several cancers and reported to be associated with increased invasiveness and poor prognosis. We examined whether there is a direct relationship between mislocalization of overexpressed CENP-A and CIN using HeLa and chromosomally stable diploid RPE1 cell lines as model systems. Our results show that mislocalization of overexpressed CENP-A to chromosome arms leads to chromosome congression defects, lagging chromosomes, micronuclei formation and a delay in mitotic exit. CENP-A overexpressing cells showed altered localization of centromere and kinetochore associated proteins such as CENP-C, CENP-T and Nuf2 leading to weakened native kinetochores as shown by reduced interkinetochore distance and CIN. Importantly, our results show that mislocalization of CENP-A to chromosome arms is one of the major contributors for CIN as depletion of histone chaperone DAXX prevents CENP-A mislocalization and rescues the reduced interkinetochore distance and CIN phenotype in CENP-A overexpressing cells. In summary, our results establish that CENP-A overexpression and mislocalization result in a CIN phenotype in human cells. This study provides insights into how overexpression of CENP-A may contribute to CIN in cancers and underscore the importance of understanding the pathways that prevent CENP-A mislocalization for genome stability.

## INTRODUCTION

Chromosomal instability (CIN), a hallmark of aggressive tumors and birth defects, is characterized by unequal distribution of chromosomes into two daughter cells (numerical CIN) and/or structural rearrangements of the genome (structural CIN). Under selective conditions, CIN results in aneuploidy, which is observed in 90% of solid tumors [1–3]. Numerical CIN is often caused by chromosome segregation errors due to mitotic abnormalities such as defective sister chromatid cohesion, over duplication of centrosomes, erroneous kinetochore-microtubule attachments and deregulation of spindle assembly checkpoint (SAC) [4]. Structural CIN ranges from small changes at the level of a few nucleotides to large structural aberrations at the chromosomal level which result in deletion, duplication or translocation of certain regions of the chromosome [4, 5].

One of the key determinants for chromosomal stability is the centromere which serves not only as a site for kinetochore assembly but also a locus for kinetochore-microtubule (KT-MT) attachments and SAC functions. Despite the differences in DNA sequence and size, nearly all eukaryotic centromeres contain an evolutionarily conserved histone H3 variant (CENP-A in human, Cse4 in budding yeast, Cnp1 in fission yeast and Cid in flies). CENP-A nucleosomes are interspersed with H3 nucleosomes along the arrays of repetitive DNA [6, 7]. CCAN (Constitutive Centromere Associated Network) proteins such as CENP-C and CENP-N specifically associate with CENP-A, whereas CENP-T and CENP-W associate with histone H3 nucleosomes [8–10]. CENP-C and CENP-T are known to have a crucial conserved role in linking centromeric chromatin to microtubules via interaction with Mis12 and the Ndc80 complex which are components of the KMN (Knl1, Mis12 and Ndc80) network, respectively [11–13]. Hence, CCAN proteins and the CENP-A nucleosome provide a platform for assembling the kinetochore and associated proteins for efficient KT-MT attachment and faithful chromosome segregation [14–16].

Strict regulation of over 80 centromere and kinetochore proteins restricts their localization to centromere or kinetochore for faithful chromosome segregation and normal cell division [15, 16]. Loading of CENP-A to centromeric chromatin is regulated by HJURP, Scm3 and Cal1 in vertebrates, budding/fission yeasts and flies, respectively [17–20]. Furthermore, inheritance of CENP-A monoubiquitination between cell divisions is also important for its deposition at the centromere and for genome stability [21–23]. Adversely, mislocalization of CENP-A to non-centromeric regions promotes aneuploidy in yeast and flies [24–29]. Moreover, defects in ubiquitin-mediated proteolysis contributes to the mislocalization of CENP-A^Cse4^ to non-centromeric regions and chromosome loss in budding yeast [30–36]. In human cells, histone H3 chaperone DAXX is required for mislocalization of overexpressed CENP-A to chromosome arms [37].

High levels of CENP-A mRNA are reported in cancers such as hepatocellular carcinoma, glioblastoma, breast cancer and several others [38–42]. CENP-A overexpression has been shown to predict poor patient survival, high risk of disease progression, higher-grade tumor, increased invasiveness and patient response to therapies [38, 42, 43]. Recently, a bioinformatics-based study showed that elevated expression of CENP-A without alteration in sequence or copy number of genomic CENP-A was observed for 20 different types of solid cancers [38]. Consistent with this observation, another study based on analysis of 12 different cancers showed that CENP-A is one of 14 centromere and kinetochore genes whose expression levels are elevated in most of these cancers [42]. Mislocalization of overexpressed CENP-A to non-centromeric regions has also been reported in cultured HeLa cells, colorectal cancer DLD1 cells and primary colorectal cancer tissues [37, 44–46]. However, the direct effect of mislocalization of overexpressed CENP-A on CIN has not yet been investigated in human cells. Given the clinical significance of CENP-A expression and tumor progression, it is critical to understand if and how CENP-A overexpression contributes to tumorigenesis and whether CENP-A expression can be exploited for prognosis, diagnosis and targeted therapy for treatment of CENP-A overexpressing cancers.

In this study, we investigate the consequences of CENP-A overexpression and mislocalization in transformed and non-transformed human cells. We provide the first evidence for a positive correlation between overexpression and mislocalization of CENP-A to CIN and show that CENP-A mislocalization is one of the major contributors for CIN. This study provides insights into how mislocalization of CENP-A may contributes to chromosome segregation defects in human cancers.

## RESULTS

### Overexpressed CENP-A mislocalizes to chromosome arms in human cells

**Figure 1:**
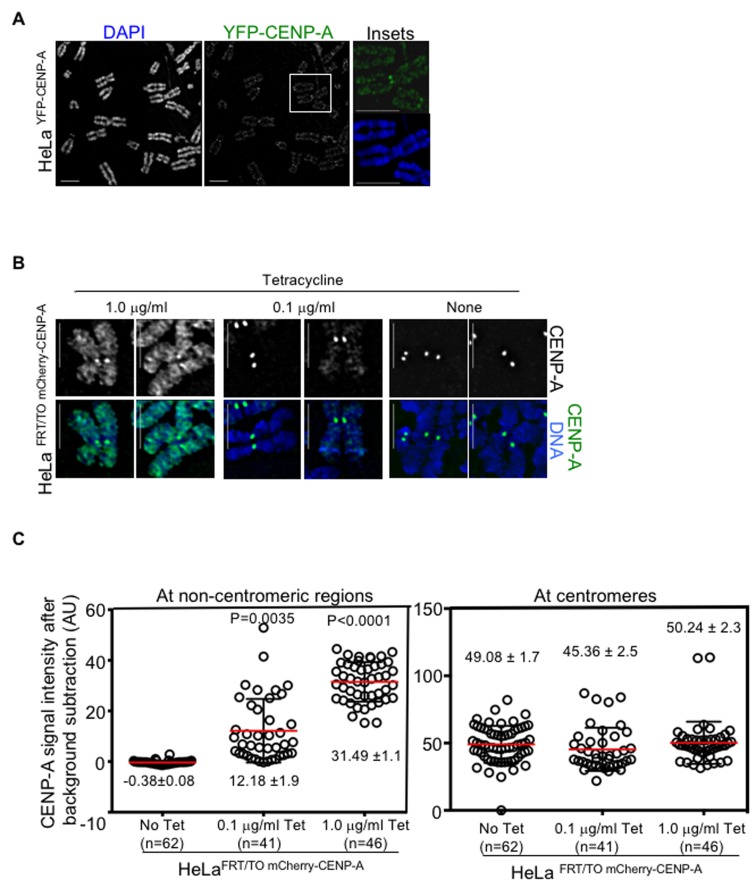
Overexpressed CENP-A mislocalizes to chromosome arms in human cells **A**. Constitutive overexpression of CENP-A leads to mislocalization to chromosome arms. Representative chromosome spread images of HeLa ^YFP-CENP-A^ cells immunostained with antibody against GFP to visualize YFP-CENP-A and stained with DAPI for DNA. Insets correspond to boxed area in the main image. Scale bar: for insets. **B**. Inducible expression of CENP-A leads to mislocalization to chromosome arms. Representative chromosome spread images of HeLa ^FRT/TO mCherry-CENP-A^ cells treated with indicated concentrations of tetracycline. Cells were immunostained with antibody against CENP-A and stained with DAPI for DNA. Scale bar: 2 μm. **C**. CENP-A levels increased at non-centromeric regions but not at centromeres in CENP-A overexpressing cells. Prism graphs show signal intensities of CENP-A at centromeres (left) and non-centromeric regions (right) in chromosome spreads of HeLa ^FRT/TO mCherry-CENP-A^ cells treated with indicated concentrations of tetracycline. Each circle represents a spot and ‘n’ denotes number of spots analyzed. Red horizontal lines represent mean signal intensity as indicated. Error bars represent standard error of mean (SEM) across areas measured. *P*-values calculated using the Mann-Whitney U test are indicated.

To study the consequences of CENP-A overexpression in human cells, a HeLa cell line constitutively overexpressing CENP-A tagged at the C-terminus with YFP (HeLa ^YFP-CENP-A^) was generated. Western blot analysis showed increased expression of YFP-CENP-A overexpression in HeLa ^YFP-CENP-A^ cells (Supplementary Figure S1A, left panel). Metaphase chromosome spreads in these cells showed mislocalization of overexpressed CENP-A in chromosome arms (Figure 1A). These results are consistent with previous observation of mislocalization of overexpressed CENP-A in HeLa cells [37, 44]. We next generated a HeLa cell line overexpressing CENP-A tagged at the C-terminus with mCherry under a tetracycline inducible promoter (HeLa ^FRT/TO mCherry-CENP-A^). This cell line permits modulation of exogenous mCherry-CENP-A expression levels by treatment with varied concentrations of tetracycline (Supplementary Figure S1A, right-upper panel). We observed no significant difference in the growth of tetracycline treated HeLa ^FRT/TO mCherry-CENP-A^ cells in comparison to untreated HeLa ^FRT/TO mCherry-CENP-A^ cells, indicating that increased CENP-A expression does not lead to cell death (Supplementary Figure S1B). As in HeLa ^YFP-CENP-A^ cells, metaphase chromosome spread analysis of HeLa ^FRT/TO mCherry-CENP-A^ cells also showed mislocalization of mCherry-CENP-A to chromosome arms following the addition of tetracycline (Figure 1B). Notably, higher expression correlated with higher mislocalization of mCherry-CENP-A (compare mislocalization of mCherry CENP-A in 1.0 μg/ml and 0.1 μg/ml tetracycline treatment) (Figure 1B). Next, we quantified the signal intensity of mCherry-CENP-A at the centromeres and non-centromeric regions in HeLa ^FRT/TO mCherry-CENP-A^ cells with and without treatments with different concentrations of tetracycline. This analysis revealed that in HeLa ^FRT/TO mCherry-CENP-A^ cells following treatment with 0.1 μg/ml and 1.0 μg/ml tetracycline, CENP-A levels at non-centromeric regions increased 10-fold and 30-fold, respectively, compared to HeLa ^FRT/TO mCherry-CENP-A^ cells without tetracycline treatment (Figure 1C, left panel). However, the level of the centromeric pool of CENP-A was not significantly affected in tetracycline treated HeLa ^FRT/TO mCherry-CENP-A^ cells when compared to untreated HeLa ^FRT/TO mCherry-CENP-A^ cells (Figure 1C, right panel). Based on these results, we conclude that both constitutive and inducible overexpression of CENP-A result in its mislocalization to chromosome arms.

### CENP-A overexpressing cells exhibit defects in chromosome congression and segregation

**Figure 2:**
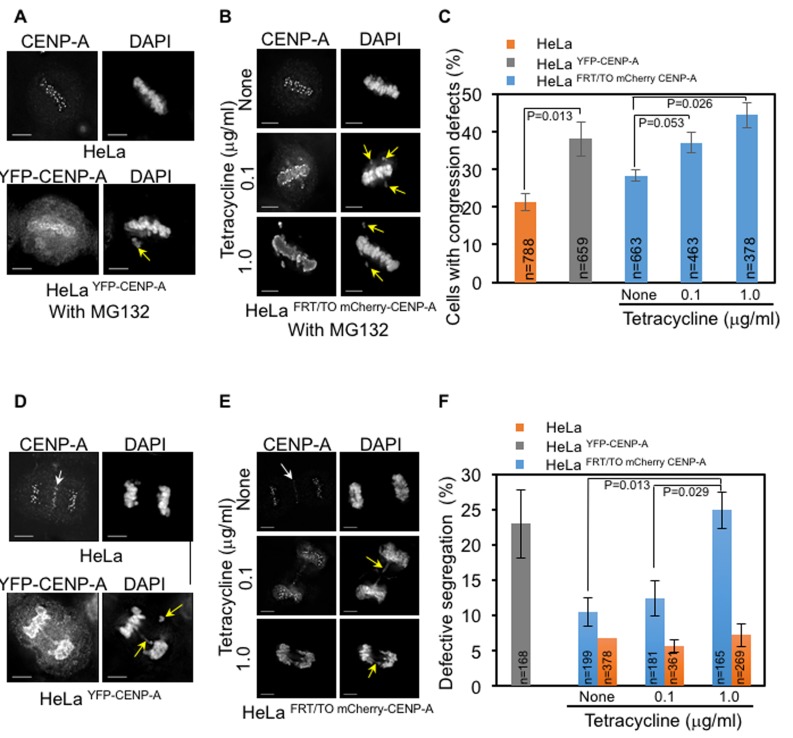
CENP-A overexpression contributes to chromosome congression and segregation defects in human cells **A**.-**B**. Constitutive and inducible expression of CENP-A contribute to chromosome congression defects. Immunofluorescence images of HeLa and Hela ^YFP-CENP-A^ (A) and HeLa ^FRT/TO mCherry-CENP-A^ (B) cells show chromosome congression status. HeLa ^FRT/TO mCherry-CENP-A^ cells were treated with different concentrations of tetracycline. Prior to immunostaining, all cells were treated with 10 μM MG132 for three hours. Cells were immunostained with antibodies against CENP-A for HeLa and HeLa ^FRT/TO mCherry-CENP-A^ and GFP for Hela ^YFP-CENP-A^ to visualize CENP-A. Cells were also stained with DAPI for DNA. Immunostained cells were imaged and analyzed for chromosome congression status. Yellow arrows show uncongressed chromosomes. Scale bar is 5 μm. **C**. Proportion of cell with defective chromosome congression is higher in CENP-A overexpressing cells. Bar chart shows the proportion of HeLa, HeLa ^YFP-CENP-A^ and HeLa ^FRT/TO mCherry-CENP-A^ cells with defective chromosome congression. Error bar represents standard error of mean (SEM) across five (for HeLa and HeLa ^YFP CENP-A^) or three (for HeLa ^FRT/TO mCherry CENP-A^) independent experiments. ‘n’ denotes number of cells analyzed. **D**.-**E**. Constitutive and inducible expression of CENP-A contribute to chromosome segregation defects. Immunofluorescence images of HeLa and Hela ^YFP-CENP-A^ (D) and HeLa ^FRT/TO mCherry-CENP-A^ (E) cells show chromosome segregation status. HeLa ^FRT/TO mCherry-CENP-A^ cells were treated with different concentrations of tetracycline. Cells were immunostained with antibodies against CENP-A for HeLa and HeLa ^FRT/TO mCherry-CENP-A^ and GFP for Hela ^YFP-CENP-A^ to visualize CENP-A. Cells were also stained with DAPI for DNA. Immunostained cells were imaged and analyzed for mitotic outcomes. White arrows show non-specific staining of CENP-A at the midzone and yellow arrows show missegregated chromosomes and DNA bridges. Scale bar: 5 μm. **F**. Proportion of cells with defective chromosome segregation is higher in CENP-A overexpressing cells. Bar chart shows the proportion of HeLa ^YFP-CENP-A^, HeLa ^FRT/TO mCherry-CENP-A^ and HeLa cells with defective segregation of chromosomes. HeLa and HeLa ^FRT/TO mCherry-CENP-A^ cells were treated with indicated concentrations of tetracycline. Error bars represent standard error of mean (SEM) from three independent experiments. ‘n’ denotes number of cells analyzed. *P*-values calculated using the proportion test are indicated.

**Figure 3:**
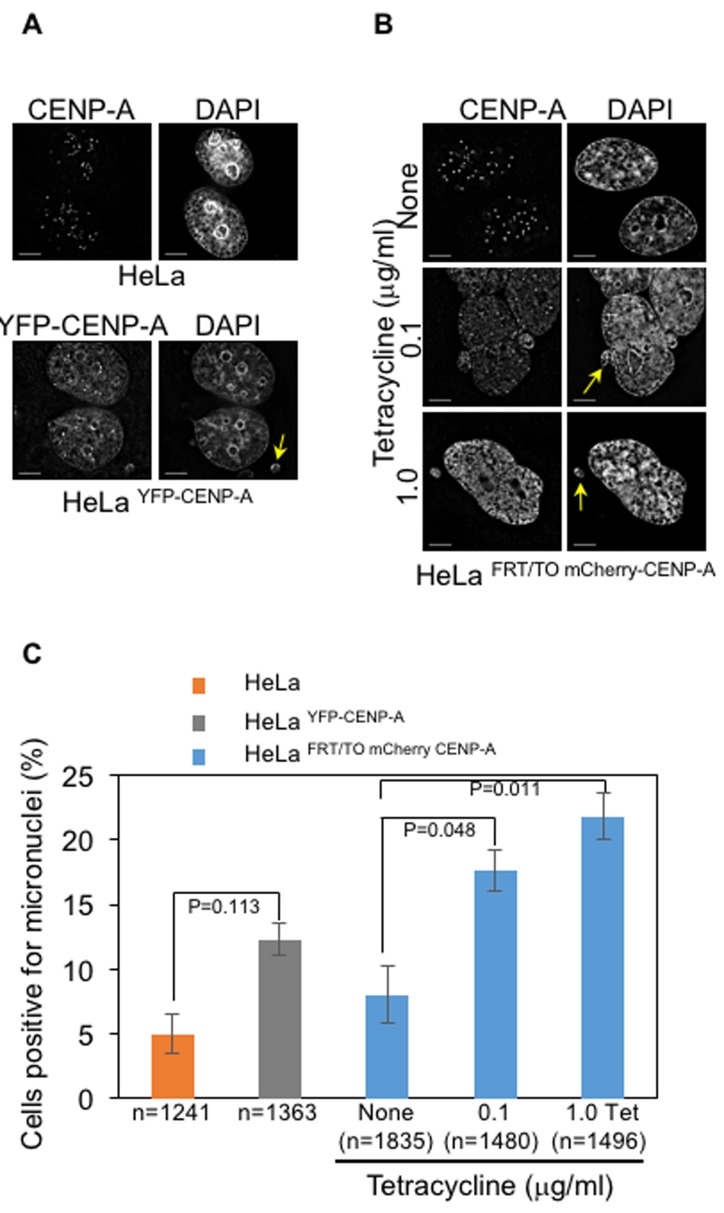
CENP-A overexpression contributes to increased incidence of micronuclei in human cells **A**.-**B**. Constitutive and inducible expression of CENP-A contribute to increased incidence of micronuclei. Immunofluorescence images of HeLa and Hela ^YFP-CENP-A^ (A) and HeLa ^FRT/TO mCherry-CENP-A^ (B) show presence of micronuclei in interphase cells. Prior to immunostaining, HeLa ^FRT/TO mCherry-CENP-A^ cells were treated with different concentrations of tetracycline. Cells were immunostained with antibodies against CENP-A for HeLa and HeLa ^FRT/TO mCherry-CENP-A^ and GFP for Hela ^YFP-CENP-A^ to visualize CENP-A. Cells were also stained with DAPI for DNA. Immunostained cells were imaged and analyzed for presence of micronuclei. Yellow arrows show micronuclei in HeLa ^YFP CENP-A^ and HeLa ^FRT/TO mCherry-CENP-A^ cells treated with different concentrations of tetracycline. Scale bar: 5 μm. **C**. Proportion of cells positive for micronuclei is higher in CENP-A overexpressing cells. Bar chart shows the proportion of HeLa, HeLa ^YFP-CENP-A^ and HeLa ^FRT/TO mCherry-CENP-A^ with micronuclei. Error bars represent standard error of mean (SEM) from three (for HeLa and HeLa ^YFP CENP-A^) or six (for HeLa ^FRT/TO mCherry CENP-A^) independent experiments. ‘n’ denotes number of cells analyzed. *P*-values calculated using the proportion test are indicated

CENP-A mislocalization and chromosome missegregation have been observed following CENP-A^Cse4^ or CENP-A^CID^ overexpression in yeasts and flies, respectively [24–30]. We investigated whether mislocalized CENP-A promotes chromosome segregation defects in human cells. We examined the consequences of CENP-A overexpression on mitosis by analyzing the status of chromosome congression in metaphase, chromosome segregation in anaphase and incidence of micronuclei. We scored for cells with defective chromosome congression- as depicted by the presence of at least one chromosome that failed to congress at metaphase plate and defective segregation- as depicted by the presence of missegregated chromosomes, lagging chromosomes and DNA bridges. Fixed and immunostained HeLa, HeLa ^YFP-CENP-A^ and HeLa ^FRT/TO mCherry-CENP-A^ cells were assayed for chromosome congression after treatment with proteasome inhibitor MG132, which arrests cells in metaphase. We observed that the proportion of cells with uncongressed chromosomes was significantly increased in HeLa^YFP-CENP-A^ and tetracycline treated HeLa ^FRT/TO mCherry-CENP-A^ cells, compared to that in HeLa cells and HeLa ^FRT/TO mCherry-CENP-A^ cells without tetracycline treatment (Figure 2A-2C). Next, we examined chromosome segregation status due to mislocalization of overexpressed CENP-A and observed that both HeLa ^YFP-CENP-A^ cells and tetracycline treated HeLa ^FRT/TO mCherry-CENP-A^ cells displayed a significant increase in the proportion of cells with defective chromosome segregation when compared to HeLa cells expressing endogenous CENP-A (Figure 2D-2F). Hence, we conclude that higher expression of CENP-A correlates with greater defects in chromosome segregation. Chromosome segregation defects in HeLa cells with and without tetracycline were not significantly different, thereby ruling out an effect of tetracycline alone on mitosis (Figure 2F). In addition to chromosome segregation defects, CIN also manifests with higher incidence of micronuclei that often results from DNA bridges due to defective chromosome segregation. HeLa ^YFP-CENP-A^ and HeLa ^FRT/TO mCherry-CENP-A^ cells treated with tetracycline showed higher proportion of cells with micronuclei (Figure 3A-3C), further supporting the conclusion that CENP-A overexpression and mislocalization contribute to CIN. We confirmed that high levels of CENP-A expression and mislocalization but not epitope tagging of CENP-A contribute to mitotic defects in cells overexpressing high levels of YFP-CENP-A. This was done by analyzing mitotic phenotypes in HeLa cells overexpressing low levels of YFP-CENP-A (HeLa^YFP-CENP-A-Low)^. CENP-A was not mislocalized in HeLa^YFP-CENP-A-Low^ cells (Supplementary Figure S2A) and the proportions of cells with uncongressed chromosomes or defective segregation in these cells were not significantly different than in HeLa cells (Supplementary Figure S2A-S2D).

Errors in chromosome segregation are monitored by the SAC and premature silencing of the SAC can contribute to mitotic exit with missegregated chromosomes. Cells with defective SAC fail to arrest in mitosis after treatment with nocodazole, a microtubule- depolymerizing agent. To identify whether defective chromosome segregation observed in CENP-A overexpressing cells is due to compromised SAC activity, we monitored the mitotic progression of HeLa ^FRT/TO cherry-CENP-A^ cells with and without tetracycline after nocodazole treatment. Analysis of over 100 cells showed that nearly all HeLa ^FRT/TO mCherry-CENP-A^ cells with or without tetracycline treatment showed an arrest in mitosis, indicating that the SAC is intact in these cells (Supplementary Figure S3A). To further confirm this, we examined the localization of MAD1, one of the SAC components that localizes to tensionless kinetochores with defective KT-MT attachments [47, 48]. Localization of MAD1 to prometaphase kinetochores in HeLa ^FRT/TO cherry-CENP-A^ cells treated with tetracycline (Supplementary Figure S3B, upper panel) supports our conclusion of an effective SAC in early mitosis in these cells. Consistent with these results, we did not observe localization of MAD1 on lagging chromosomes in HeLa ^FRT/TO mCherry-CENP-A^ cells with or without tetracycline treatment undergoing anaphase (Supplementary Figure S3B, lower panel). Collectively, these results show that chromosome segregation defects in CENP-A overexpressing cells are not due to defects in SAC and premature mitotic exit. Therefore, our results on the dose-dependent effect of CENP-A overexpression and mislocalization on chromosome segregation support our conclusion that mislocalization of CENP-A leads to a CIN phenotype in human cells.

### CENP-A overexpression induces mislocalization in chromosomally stable diploid RPE1 cells

**Figure 4:**
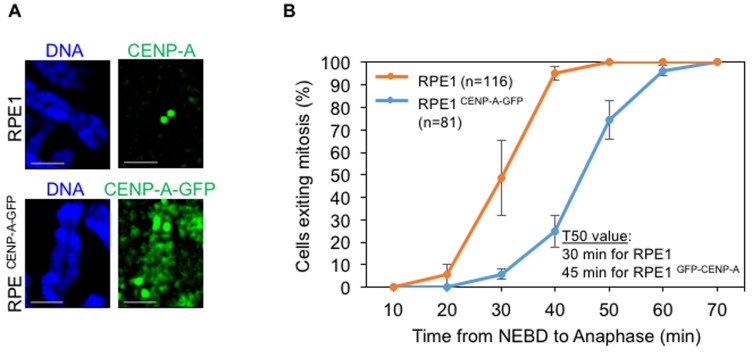
CENP-A overexpression induces mislocalization in non-transformed diploid RPE1 cell line **A**. Overexpressed CENP-A mislocalizes to chromosome arms in RPE1 cells. Representative images of chromosomes of RPE1 and RPE1 ^CENP-A-GFP^ cells show localization of CENP-A. Chromosome spreads were prepared and immunostained with antibodies against CENP-A for RPE1 and GFP for RPE1 ^CENP-A-GFP^ to visualize CENP-A and stained with DAPI for DNA. Scale bar: 1 μm. **B**. CENP-A overexpressing RPE1 cells show a delay in mitotic exit. Cumulative distribution graph of mitotic exit time (from Nuclear envelop breakdown to anaphase) in RPE1 and RPE1 ^CENP-A-GFP^ cells accrued from time-lapse movies. GFP and DIC channels for RPE ^CENP-A GFP^ cells and DIC alone for RPE1 cells were used to ascertain the nuclear envelope breakdown (NEBD) and anaphase onset. T50 represents the time point at which at least 50% of cells exited mitosis. ‘n’ denotes number of cells analyzed. Error bars represent standard error of mean from four independent experiments.

HeLa cells are transformed and chromosomally unstable, hence we examined the consequences of CENP-A overexpression in a non-transformed chromosomally stable diploid RPE1 cell line. An RPE1 cell line constitutively overexpressing CENP-A-GFP (RPE1 ^CENP-A-GFP^) was generated to examine the localization of overexpressed CENP-A in chromosome spreads. We determined that overexpressed CENP-A mislocalized to chromosome arms in RPE1 ^CENP-A-GFP^ cells (Figure 4A), similar to that observed in HeLa ^YFP-CENP-A^ and tetracycline treated HeLa ^FRT/TO mCherry-CENP-A^ cells. Time-lapse analysis for the mitotic timing of RPE1 ^CENP-A-GFP^ cells revealed that these cells underwent delayed mitosis (T_50_ = 45 min, n=81) when compared to RPE1 cells without CENP-A overexpression (T_50_ = 30 min, n=116) (Figure 4B). This data further shows that chromosome segregation errors observed in CENP-A overexpressing cells are not due to premature mitotic exit. Taken together, our results show that overexpressed CENP-A mislocalizes to chromosome arms and induces mitotic defects in both transformed (HeLa) and non-transformed (RPE1) cell lines.

### Levels of a subset of kinetochore proteins are reduced at centromeres in CENP-A overexpressing cells

**Figure 5:**
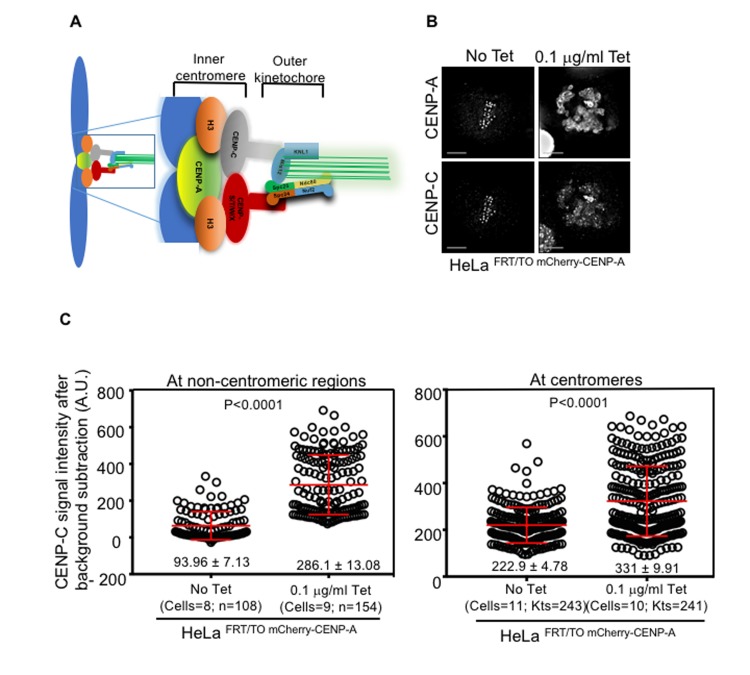
CENP-C mislocalizes to non-centromeric regions in CENP-A overexpressing cells **A**. Schematic diagram showing subsets of outer and inner kinetochore proteins. **B**. CENP-C mislocalizes to non-centromeric regions in CENP-A overexpressing cells. Representative immunofluorescence images of HeLa ^FRT/TO mCherry-CENP-A^ cells untreated or treated with 0.1 μg/ml tetracycline show mislocalization of CENP-C along with overexpressed CENP-A. Cells were immunostained with antibodies against CENP-C, CENP-A, and CREST antisera for centromeres. Scale bar is 5 μm. **C**. CENP-C levels increase at centromeres and non-centromeric regions in CENP-A overexpressing cells. Prism graphs show CENP-C signal intensities at non-centromeric regions (left panel) and at centromeres (right panel) in the metaphase plate of HeLa ^FRT/TO mCherry-CENP-A^ cells untreated or treated with 0.1 μg/ml of tetracycline. Each circle represents a spot or a centromere and ‘n’ or ‘Kts’ denotes number of spots or centromeres analyzed in certain number of cells as denoted by ‘cells’. Red horizontal lines represent mean signal intensities as indicated. Error bars represent standard error of mean (SEM) across the kinetochores measured from at least two independent experiments. *P*-values calculated using the Mann-Whitney U test are indicated.

CENP-A plays an important role in functional kinetochore assembly by recruiting other components of the CCAN such as CENP-C and CENP-S/T/W/X. These centromeric proteins form a bridge with outer kinetochore proteins of the KMN network where microtubules attach for proper segregation of chromosomes (Figure 5A) [11, 49, 50]. Moreover, CENP-C forms a stable complex with CENP-A nucleosomes and stabilizes centromeric chromatin [51, 52]. CENP-C has been shown to mislocalize in cells overexpressing CENP-A [37, 44], hence we examined whether CENP-C mislocalizes to non-centromeric regions in our cell line model system. We found that endogenous CENP-C significantly mislocalized upon induction of CENP-A in HeLa ^FRT/TO mCherry-CENP-A^ cells treated with tetracycline (Figure 5B). Quantitative analysis of CENP-C signal intensity revealed higher levels of CENP-C at both non-centromeric and centromeric regions in tetracycline treated HeLa ^FRT/TO mCherry-CENP-A^ cells compared to untreated HeLa ^FRT/TO mCherry-CENP-A^ cells (Figure 5C). Next, we examined if the expression of CENP-C was increased in cells overexpressing CENP-A. Western blot analysis showed that expression of CENP-C was not significantly altered in whole cell lysates of HeLa ^FRT/TO mCherry-CENP-A^ cells with or without tetracycline treatment (Supplementary Figure S1A, right-lower panel).

**Figure 6:**
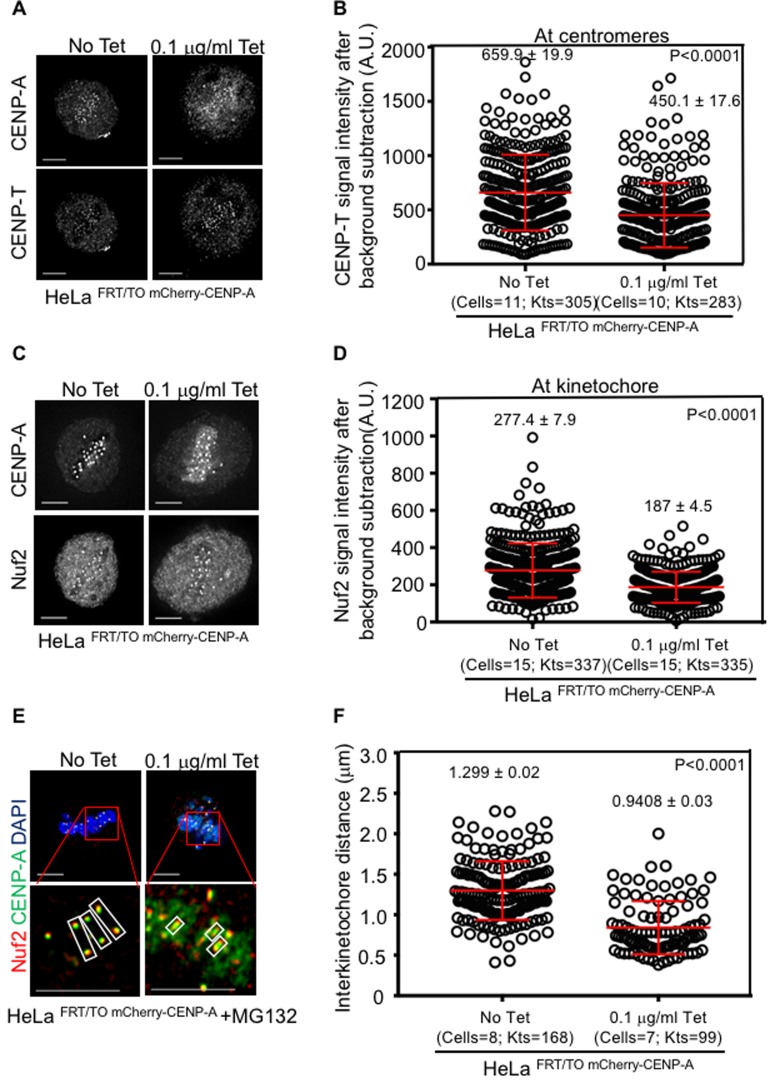
Reduced levels of CENP-T and Nuf2 at kinetochores contribute to reduced interkinetochore distance in CENP-A overexpressing cells **A**. and **C**. CENP-T and Nuf2 do not mislocalize in CENP-A overexpressing cells. Representative immunofluorescence images of HeLa ^FRT/TO mCherry-CENP-A^ cells untreated or treated with 0.1 μg/ml tetracycline show normal localization of CENP-T (A) and Nuf2 (C), along with CENP-A. Cells were immunostained with indicated antibodies. Scale bar is 5 μm. **B**. and **C**. CENP-T and Nuf2 levels are reduced at the centromere and kinetochore, respectively in CENP-A overexpressing cells. Prism graphs show signal intensities of CENP-T at centromeres (B) and Nuf2 at kinetochores **D**. in metaphase plates of HeLa ^FRT/TO mCherry-CENP-A^ cells untreated or treated with 0.1 μg/ml of tetracycline. Each circle represents each centromere (for B) or kinetochore (for D) and ‘Kts’ denotes number of centromeres or kinetochores analyzed in certain number of cells as denoted by ‘cells’. **E.** Interkinetochore distance is reduced in CENP-A overexpressing cells. Representative immunostained images of HeLa ^FRT/TO mCherry-CENP-A^ cells untreated or treated with 0.1 μg/ml of tetracycline show interkinetochore distance. Prior to immunostaining, cells were treated with 10 μM MG132 for 3 hours. Cells were then immunostained with antibodies against CENP-A, Nuf2 as outer kinetochore marker, CREST antisera for centromeres and stained with DAPI for DNA. Insets correspond to red boxed areas in main images. White boxed areas in insets show examples for kinetochore pairs included in analysis. Scale bar is 5 μm. **F**. Prism graph shows interkinetochore distance ascertained by distance between two Nuf2 signals within aligned chromosomes in MG132-arrested HeLa ^FRT/TO mCherry-CENP-A^ cells untreated or treated with 0.1 μg/ml tetracycline. Each circle represents each kinetochore pair and ‘Kts’ denotes number of kinetochore pairs analyzed in certain number of cells as denoted by ‘cells’. Red horizontal lines represent mean signal intensities (B and D) or mean interkinetochore distance (F) as indicated. Error bars represent standard error of mean (SEM) across kinetochores measured from at least two independent experiments. *P*-values calculated using the Mann-Whitney U test are indicated.

We next examined the localization of DNA-binding protein CENP-B and CENP-C interacting partners, Mis12 and CENP-T, in CENP-A overexpressing cells. Obvious mislocalization of CENP-B or Mis12 to non-centromeric regions was not observed and levels of Mis12 at the kinetochores were similar between HeLa ^FRT/TO mCherry-CENP-A^ cells with or without tetracycline treatment (Supplementary Figure S4A-S4C). In contrast, signal intensity measurement of CENP-T at the centromere revealed that levels of centromere associated CENP-T were significantly reduced (57% reduction compared to control) without any obvious mislocalization to non-centromeric regions in HeLa ^FRT/TO cherry-CENP-A^ cells treated with tetracycline (Figure 6A and 6B). Reduced levels of CENP-T at the centromere prompted us to examine the localization of outer kinetochore protein Nuf2, a component of Ndc80 complex interacting with CENP-T. Consistent with observations for CENP-T, Nuf2 mislocalization to non-centromeric regions was not observed but its signal intensity levels at kinetochores were reduced (40% reduction compared to control) in HeLa ^FRT/TO cherry-CENP-A^ cells treated with tetracycline (Figure 6C and 6D). Taken together, our results show that mislocalization of CENP-A contributes to mislocalization of CENP-C to non centromeric regions and reduced levels of CENP-T and Nuf2 at the centromeres and kinetochores, respectively.

### Mislocalization of CENP-A contributes to weakened native kinetochores in CENP-A overexpressing cells

Reduced levels of CCAN components, CENP-T and outer kinetochore protein Nuf2 at centromere and kinetochore led us to postulate that CENP-A overexpression may weaken the native kinetochore. We investigated the strength of native kinetochores in HeLa ^FRT/TO mCherry-CENP-A^ cells with or without tetracycline treatment by measuring the distance between two kinetochores (interkinetochore distance) in metaphase. The rationale behind this is, proper KT-MT attachment generates a strong pulling force on sister chromatids towards opposing spindle poles creating a certain distance between two kinetochores [53, 54]. Therefore, interkinetochore distance between two kinetochores of sister chromatids can be used as a proxy for kinetochore strength. Interkinetochore distance was examined in MG132 arrested HeLa ^FRT/TO mCherry-CENP-A^ metaphase cells with and without tetracycline treatment by measuring the distance between Nuf2 foci on each sister chromatid within an aligned chromosome. Significant reduction in distance between Nuf2 signals on aligned sister chromatids was observed in tetracycline treated HeLa ^FRT/TO mCherry-CENP-A^ cells (0.940 ± 0.03 μm) compared to that in HeLa ^FRT/TO mCherry-CENP-A^ cells without tetracycline treatment (1.299 ± 0.02 μm) (Figure 6E and 6F). Similar results for reduced interkinetochore distance were also observed when distance between CENP-A signals on two aligned sister chromatids was measured in tetracycline treated HeLa ^FRT/TO mCherry-CENP-A^ cells (0.88 ± 0.02 μm) compared to that in HeLa ^FRT/TO mCherry-CENP-A^ cells without tetracycline treatment (1.02 ± 0.02 μm) (Supplementary Figure S5A and S5B). These results support our hypothesis that CENP-A overexpression weakens the native kinetochore and contributes to a CIN phenotype in CENP-A overexpressing cells.

### Suppressing mislocalization of CENP-A rescues CIN phenotype and reduced interkinetochore distance in CENP-A overexpressing cells

**Figure 7:**
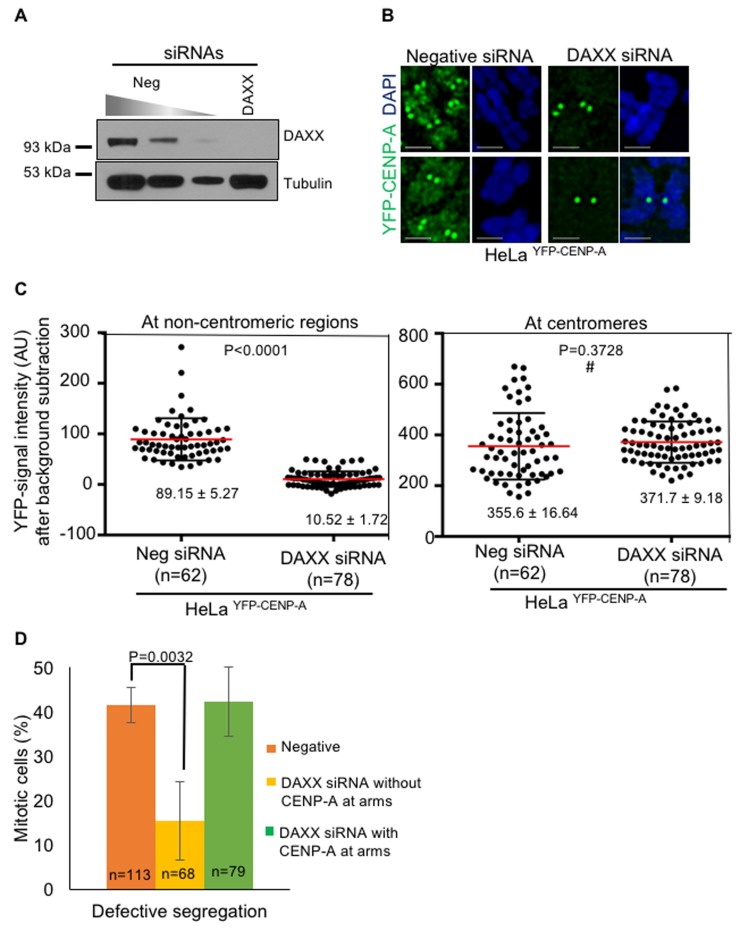
Depletion of DAXX prevents mislocalization of CENP-A and rescues chromosome segregation defects in CENP-A overexpressing cells **A**. DAXX is efficiently depleted in DAXX siRNA-treated cells. Immunoblot shows DAXX levels in cell lysates treated with siRNAs as indicated. HeLa ^YFP-CENP-A^ cells were treated with siRNAs as indicated for 72 hours. Cell lysates were collected using 2x laemmli buffer for western blot analysis. Blots were probed with anti-DAXX Ab to assess depletion efficiency of DAXX following treatment with DAXX siRNA oligo. Anti-tubulin Ab was used as a loading control. **B**. DAXX depletion prevents CENP-A mislocalization. Representative images of chromosomes show expression and localization of YFP-CENP-A in HeLa ^YFP-CENP-A^ cells treated with siRNAs as indicated. Chromosome spreads were prepared and immunostained with antibody against GFP to visualize CENP-A and stained with DAPI for DNA. Cells were then imaged and analyzed for YFP-CENP-A localization. Scale bar: 1 μm. **C**. CENP-A levels are reduced in non-centromeric regions following DAXX depletion. Prism graphs show CENP-C signal intensities at non-centromeric regions (left) and at centromeres (right) in chromosome spreads of HeLa ^YFP-CENP-A^ cells treated with siRNAs as indicated. Each circle represents a spot and ‘n’ denotes number of spots analyzed. Red horizontal lines represent mean signal intensities as indicated. Error bars represent standard error of mean (SEM) across the areas measured. *P*-values calculated using the Mann-Whitney U test are indicated. # represents statistically insignificant values. **D**. DAXX depletion suppresses chromosome segregation defects. Bar chart graph shows proportion of HeLa ^YFP-CENP-A^ cells with defective chromosome segregation in different conditions. Error bar represents standard error of mean across three independent experiments. ‘n’ denotes number of cells analyzed. P value calculated using proportion test is indicated.

We examined whether restricting the localization of CENP-A to the centromere suppresses the CIN phenotype in CENP-A overexpressing cells. The histone chaperone DAXX is required for CENP-A mislocalization [37]. Hence, we examined if a knockdown of DAXX would suppress the CIN phenotype in CENP-A overexpressing cells. First, we examined whether DAXX depletion rescues CENP-A mislocalization to chromosome arms in HeLa ^YFP-CENP-A^ cell line. We used RNAi to deplete DAXX and western blot analysis confirmed efficient depletion of DAXX in HeLa ^YFP-CENP-A^ cells treated with DAXX siRNA oligos (Figure 7A). Consistent with previous results [37], chromosome spreads analysis showed reduced CENP-A in chromosome arms in DAXX siRNA treated HeLa ^YFP-CENP-A^ cells compared to the negative siRNA treated HeLa ^YFP-CENP-A^ cells (Figure 7B). Quantification of CENP-A signal intensity further confirmed that non-centromeric CENP-A in DAXX depleted HeLa ^YFP-CENP-A^ cells was significantly reduced (10.52±1.72 A.U.) compared to high levels in negative siRNA treated HeLa ^YFP-CENP-A^ cells (89.15±5.2 A.U.) (Figure 7C, left panel). In contrast, centromeric levels of CENP-A were unchanged in both DAXX siRNA and negative siRNA treated HeLa ^YFP-CENP-A^ cells (355.6± 16.64 A.U. and 371.7±9.18 A.U., respectively) (Figure 7C, right panel).

We next asked whether DAXX depletion could suppress chromosome segregation defects induced by CENP-A overexpression. Mitotic HeLa ^YFP-CENP-A^ cells treated with DAXX siRNA or negative siRNA oligos were examined for errors in chromosome segregation. Consistent with our earlier observations (Figure 2D-2F), a significant proportion of CENP-A overexpressing cells (n=113) treated with negative siRNA oligo exhibited defects in chromosome segregation, as ascertained by the presence of missegregated chromosomes, lagging chromosomes and DNA bridges (40%). Strikingly, DAXX siRNA treated cells that do not show CENP-A in chromosome arms (n=68) showed reduced chromosome segregation errors (15%) (Figure 7D). In DAXX siRNA treated cells in which CENP-A mislocalization was not suppressed (n=79, 15% of the total cells), chromosome segregation errors were comparable to that observed in negative siRNA treated cells (40%). Based on these results, we conclude that CENP-A mislocalization to non-centromeric regions contributes to a CIN phenotype in CENP-A overexpressing cells.

**Figure 8:**
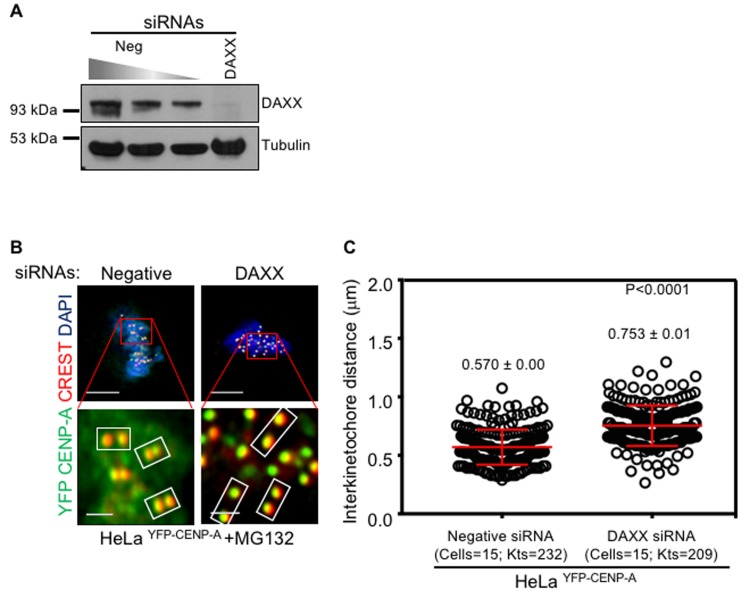
Depletion of DAXX rescues reduced interkinetochore distance in CENP-A overexpressing cells **A**. DAXX is efficiently depleted in DAXX siRNA-treated cells. Immunoblot shows DAXX levels in cell lysates treated with siRNAs as indicated. HeLa ^YFP-CENP-A^ cells were treated with siRNAs as indicated for 72 hours. Cell lysates were collected using 2x laemmli buffer for western blot analysis. Blots were probed with anti-DAXX Ab to assess depletion efficiency of DAXX following treatment with DAXX siRNA oligo. Anti-tubulin Ab was used as a loading control. **B**. DAXX depletion rescues reduced interkinetochore distance in CENP-A overexpressing cells. Representative immunostained images of HeLa ^YFP CENP-A^ cells treated with negative or DAXX siRNA oligos. Prior to immunostaining, cells were treated with 10 μM MG132 for 3 hours. Cells were then immunostained with antibodies against CENP-A, CREST antisera for centromeres and stained with DAPI for DNA. Insets correspond to red boxed areas in main images. White boxed areas in insets show examples of kinetochore pairs included in analysis. **C**. Prism graph shows interkinetochore distance ascertained by distance between two CENP-A signals within aligned chromosomes in MG132-arrested HeLa ^YFP CENP-A^ cells treated with negative or DAXX siRNA oliogs. Each circle represents a kinetochore pair and ‘Kts’ denotes number of kinetochore pairs analyzed in certain number of cells as denoted by ‘cells’. Red horizontal lines represent mean interkinetochore distance as indicated. Error bars represent standard error of mean (SEM) across kinetochores measured from at least two independent experiments. *P*-values calculated using the Mann-Whitney U test are indicated.

Interkinetochore distance was reduced in CENP-A overexpressing cells (Figure 6E and 6F) due to weakened kinetochores resulting in chromosome segregation defects. Hence, we examined whether the interkinetochore distance increases upon depletion of DAXX in CENP-A overexpressing cells. Western blot analysis confirmed that treatment with DAXX siRNA oligo efficiently depleted DAXX (Figure 8A). We measured the distance between centromeric CENP-A foci on each sister chromatid within an aligned chromosome in MG132 arrested HeLa ^YFP-CENP-A^ metaphase cells treated with negative or DAXX siRNA oligos. Quantitative analysis revealed that interkinetochore distance was reduced in HeLa ^YFP-CENP-A^ cells treated with negative siRNA oligo (0.57 ± 0.0 μm) whereas it was increased in HeLa ^YFP-CENP-A^ cells treated with DAXX siRNA oligo (0.75 ± 0.01 μm) (Figure 8B and 8C). Hence, we conclude that preventing mislocalization of CENP-A suppresses the CIN phenotype that is due to weakened kinetochores in CENP-A overexpressing cells.

## DISCUSSION

**Figure 9:**
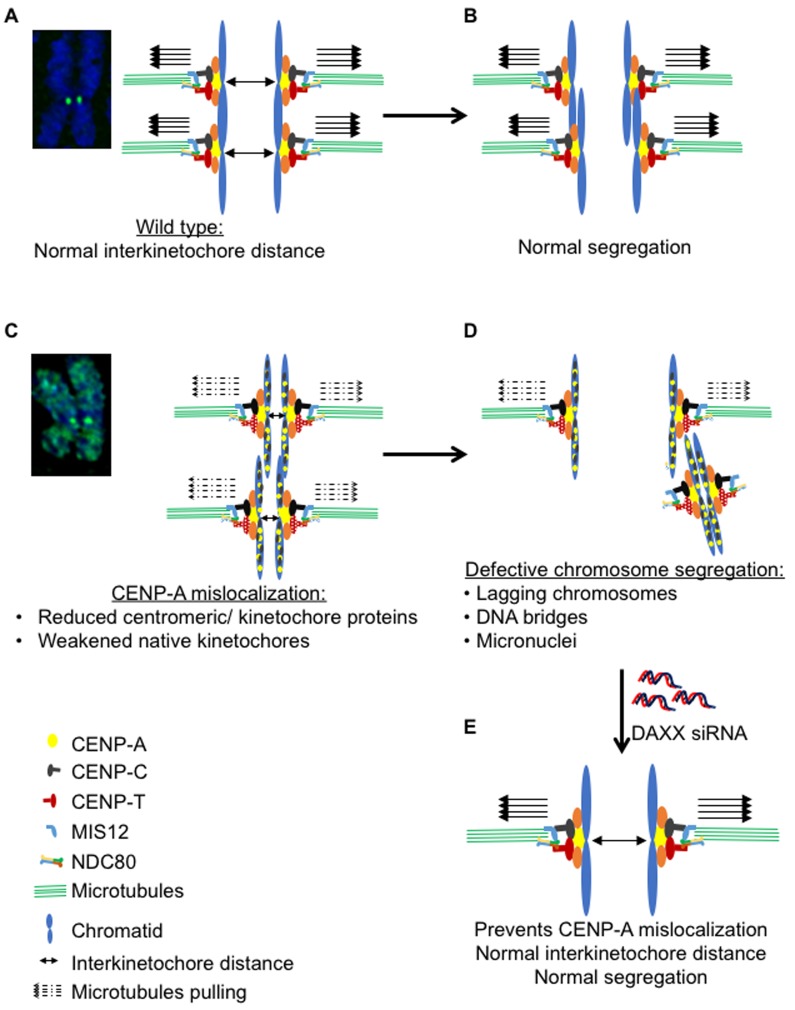
Model illustrating mislocalization of CENP-A contributes to a CIN phenotype In wild type cells, functional kinetochores provide efficient pulling forces (solid arrows) for separation of sister chromatids during metaphase to anaphase (**A**) thereby ensuring normal segregation (**B**). Mislocalization of CENP-A results in reduced levels of CENP-T and Nuf2, at kinetochores thereby weakening of the native kinetochores as reflected by the reduced interkinetochore distance (**C**) and leading to defective chromosome segregation (**D**). Depletion of DAXX prevents the mislocalization of CENP-A and rescues chromosome segregation defects in CENP-A overexpressing cells (**E**).

In this study, we established cell lines and developed cell biology assays to address a long-standing question: whether mislocalization of overexpressed CENP-A contributes to CIN which is observed in many cancers. Constitutive or inducible expression of CENP-A in HeLa and RPE1 cells result in mislocalization of CENP-A to non-centromeric regions. We performed a comprehensive analysis to assay the effects of CENP-A mislocalization on mitosis. Our results show a dose-dependent effect of CENP-A overexpression on chromosome segregation defects and higher incidence of micronuclei. CENP-A overexpresssing cells show altered levels of a subset of centromere and kinetochore associated proteins. Most importantly, we show that mislocalization of CENP-A is the major contributor for a CIN phenotype as preventing mislocalization of CENP-A to chromosome arms suppresses CENP-A overexpression induced chromosome segregation defects. We propose a model in which overexpression and mislocalization of CENP-A reduce the levels of a subset of centromere and kinetochore associated proteins, thereby weakening the native kinetochores and eventually leading to a CIN phenotype (Figure 9). Our results establish that overexpression and mislocalization of CENP-A contributes to chromosome segregation defects and provides insights into how CENP-A overexpression may contribute to aneuploidy in human cancers.

Although mislocalization of overexpressed CENP-A to non-centromeric regions was previously reported in HeLa and colorectal cancer cell line DLD-1 [37, 44, 45], the effect of this mislocalization on aneuploidy remained undetermined. Using an inducible expression system in which the expression of exogenous CENP-A can be modulated, we show that overexpression of CENP-A leads to mislocalization of CENP-A to chromosome arms. Furthermore, there is a strong correlation between CENP-A overexpression, mislocalization and chromosome segregation defects in HeLa cells. Higher incidence of micronuclei observed in CENP-A overexpressing cells supports the presence of lagging and missegregated chromosomes as these have been previously shown to contribute in micronuclei formation [55, 56]. Single cell sequencing has shown that micronuclei can generate chromothripsis which is characterized by massive genomic rearrangements [55]. Based on our data, we propose that CENP-A overexpression and mislocalization may contribute to aneuploidy in CENP-A overexpressing cancers. Support for this conclusion is derived from high levels of CENP-A mRNA in chromosomally instable cancers and chromosome segregation defects after transient transfection of CENP-A-RFP in HeLa cells [20, 42].

We hypothesize that chromosome segregation defects in CENP-A mislocalized cells may be because these cells: a) fail to activate the SAC, b) have an altered transcriptome, c) form functional neocentromeres at ectopic sites, or d) exhibit defects in structure and function of the endogenous kinetochore. To address the first possibility, we checked the status of MAD1 which localizes to kinetochores when SAC is active [47, 57] and the SAC activity by examining the ability of cells to arrest in mitosis after nocodazole treatment. Our results show that MAD1 localizes to uncongressed chromosomes in CENP-A overexpressing cells. Furthermore, nocodazole treated CENP-A overexpressing cells arrest in mitosis. These two lines of evidence allow us to conclude that the SAC remains active in CENP-A overexpressing cells. The second possibility predicts that mislocalization of CENP-A alters the transcriptome of the cell and this contributes to CIN in CENP-A overexpressing cells. This possibility is unlikely as a study aimed at understanding the effect of CENP-A mislocalization on histone dynamics conclusively showed that constitutive overexpression of CENP-A in HeLa cells did not affect the transcriptome [37]. The third possibility suggests that formation of neocentromeres in CENP-A overexpressing cells contributes to CIN. Previous studies have shown that cells that mislocalize overexpressed CENP-A also show mislocalization of CENP-C to non-centromeric regions [37, 44, 45]. Consistent with these results, we observed mislocalization of CENP-C, but not CENP-T, Mis12 and Nuf2 to the non-centromeric regions in CENP-A overexpressing cells. Because we failed to detect outer kinetochore proteins and CENP-T at non-centromeric regions, unlike in flies [24], we see no evidence in our system for functional neocentromeres formation following CENP-A overexpression. However, it is possible that the sensitivity of our assays precludes the detection of mislocalized CENP-T and Nuf2 to non-centromeric regions in CENP-A overexpressing cells or neocentromeres form only in a specific genetic background or cell type.

Finally, we propose the hypothesis that overexpression and mislocalization of CENP-A alters the structure and function of the endogenous kinetochore which leads to a CIN phenotype (Figure 9). Reduced levels of centromere associated CENP-T and kinetochore associated Nuf2 in CENP-A overexpressing cells support this hypothesis. CENP-T is a constitutive DNA binding centromeric component and serves as a structural platform for outer kinetochore assembly by binding with Spc24/25 of the Ndc80 complex [58]. Centromeric levels of Ndc80 are greatly reduced in CENP-T deficient cells and depletion of CENP-T contributes to chromosome segregation defects [8, 58, 59]. Reduced inter kinetochore distance due to a weakened endogenous kinetochore has been reported in cells depleted of Nuf2, a component of the Ndc80 complex and in cells with abrogation of the Ndc80 complex [60, 61]. Hence, we conclude that reduced levels of CENP-T contribute to reduced levels of centromeric Nuf2 and a weakening of the endogenous kinetochore resulting in a CIN phenotype in cells with mislocalization of overexpressed CENP-A. This is consistent with the effects of loss of CENP-A methylation which also weaken centromeres through loss of CENP-T and CENP-I, resulting in chromosome missegregation [62]. This hypothesis is supported by reduced inter kinetochore distance observed in cells with CENP-A overexpression and mislocalization. We propose a model in which overexpression and mislocalization of CENP-A reduce the levels of a subset of proteins at the centromere and kinetochore which weakens the native kinetochore and leads to a CIN phenotype (Figure 9).

We provide experimental evidence to support the conclusion that mislocalization of CENP-A is the major contributor for chromosome segregation defects. Although HJURP is a specific chaperone for recruitment of CENP-A to centromeres [18], it is not required for mislocalization of overexpressed CENP-A [37, 45]. Hence, we exploited depletion of DAXX that was previously shown to be required for mislocalization of overexpressed CENP-A to chromosome arms [37]. DAXX depletion suppressed the mislocalization of overexpressed CENP-A to chromosome arms and this correlated with rescue of reduced interkinetochore distance and chromosome segregation defects (Figures 7 and 8). Moreover, DAXX depletion did not affect the centromeric pool of CENP-A or cell viability. These results suggest that DAXX depletion may have a distinct advantage to prevent aneuploidy resulting from CENP-A overexpression and mislocalization. Several observations support this proposal as mutations in DAXX predicts better prognosis of pancreatic neuroendocrine tumors [63]. Overexpression and depletion of DAXX correlate with an enhancement or suppression of ovarian cancer cell proliferation, respectively [64]. Furthermore, bioinformatics analysis showed high levels of DAXX expression in metastatic pancreatic cancer which correlates with decreased patient survival with low levels of expression in normal prostate glands [65]. Our results showing that DAXX depletion rescues CENP-A overexpression induced chromosome missegregation provide an exciting avenue for DAXX as a biomarker for CENP-A overexpressing cancers.

In summary, this study establishes that overexpression and mislocalization of CENP-A contribute to CIN in human cells by weakening the strength of the native kinetochores due to reduced levels of a subset of proteins at the centromere and kinetochore (Figure 6A-6D). We provide evidence for CENP-A mislocalization as one of the major contributors for CIN as DAXX depletion suppresses CENP-A mislocalization, overexpression induced CIN and weakened kinetochores (Figure 7 and 8). Our results provide mechanistic insights into how CENP-A overexpression may contribute to aneuploidy in CENP-A overexpressing cancers. Characterization of pathways that prevent mislocalization of overexpressed CENP-A and identification of genes that exhibit synthetic lethality with CENP-A overexpression will aid in prognosis, diagnosis and treatment of CENP-A overexpressing cancers.

## MATERIALS AND METHODS

### Cell culture

All cell lines were cultured at 37°C with 5% CO_2_ supply in Dulbecco's modified Eagle's Media (DMEM) (12491023), except for the RPE1 cells that were cultured in DMEM/F-12 Ham media (21331020). Both media were supplemented with 10% fetal calf serum (FSC) (Sigma-F6178-500ml), penicillin/streptomycin (15140122), fungizone (15290018) and L-glutamine (A2916801). Stock cells were mixed in freezing media (DMEM with 50% FCS and 5% DMSO) and stored at -80°C. All reagents were purchased from Thermofisher Scientific unless otherwise stated. For tetracycline induction, cells were treated with the stated concentrations of tetracycline for 24 hours. For the chromosome congression assay, cells were treated with 10 μM MG132 for 3 hours.

### Generation of cell lines overexpressing CENP-A

CENP-A-GFP and YFP-CENP-A plasmids were constructed by cloning into the BamH1/EcoR1 or SnaB1 sites of the pBABE-blasticidin vector, respectively. CENP-A, eGFP and eYFP were PCR amplified and fused with the digested vectors using cold fusion (System Biosciences).

293GP retrovirus cells were co-transfected with a VSV-G plasmid and pBabe-CENP-A-GFP or pBabe-YFP-CENP-A. 24 hours after transfection, media was changed and viruses were collected at 48 and 72 hours. RPE1 and HeLa cells were infected with the virus, and positive cells were selected with 12 μg/ml blasticidin. Initially, all colonies were mixed for a heterogeneous population of positive cells, followed by FACS sorting to obtain a homogenous population of RPE1 ^CENP-A-GFP^ and HeLa ^YFP-CENP-A^ cells. From the heterogeneous population of YFP positive HeLa cells, cells overexpressing low levels of YFP were further FACS sorted to obtain HeLa YFP-CENP-A overexpressing low levels of YFP-CENP-A (HeLa ^YFP-CENP-A low^). To generate the Hela ^FRT/TO mCherry-CENP-A^ cell line, CENP-A was first amplified by PCR and cloned into the pcDNA5-FRT/TO vector containing an N-terminal mCherry tag. The HeLa ^FRT/TO mCherry-CENP-A^ cell lines were generated by FRT mediated recombination according to Invitrogen protocols, and clones were selected using 200 μg/ml Hygromycin. All cell lines were tested for mycoplasma using Universal mycoplasma detection kit (ATCC-30-1012K) according to the manufacturer's instructions.

### Chromosome spread preparation

For CENP-A localization studies, chromosome spreads were prepared as follows: Cells were treated with 0.1 μg/ml of colcemid (Roche-10295892001) for 4-6 hours, followed by mitotic shake-off. Mitotic cells were then treated with hypotonic solution, 0.8% sodium citrate, and incubated at 37°C for 20 minutes. After incubation, cells were centrifuged at 1200 RPM for 5 minutes and the supernatant was removed. The cell pellet was mixed with 0.8% sodium citrate and an appropriate volume of mixed cells was added into the cytospin funnel and spun at 900 RPM for 5 minutes. Cells were then fixed with 4% PFA, followed by permeabilization with 0.1% triton-X and analyzed in immunofluorescence.

### siRNA

All siRNA transfections were performed using Lipofectamine RNAi Max reagent (Thermofisher scientific-13778075) according to the manufacturer's instructions. For RNAi, siRNA oligos and transfecting reagent were diluted in Opti-MEM reduced serum media (Thermofisher scientific-31985088). siRNA oligo against DAXX (CTGGAACCTGGCAAACAGAT) was ordered from Dharmacon. For control-siRNA, negative control oligo (12935-300, Invitrogen) was used.

### Immunostaining and immunoblotting

For immunostaining, cells were fixed either with ice-cold methanol for 1 minute or with 4% PFA for 5 minutes, followed by permeabilization with 0.1% Triton-X for 5 minutes. Fixation was followed by blocking with 1% BSA in PBS/0.1% Tween (PBST) for 30 minutes at room temperature. Coverslips with cells were incubated in primary antibodies for 1 hour, washed 3 times in PBST and incubated with secondary antibodies for 45 minutes at room temperature. Cells were then stained with DAPI and mounted on slides using Prolong gold antifade mountant (Thermofisher scientific-P36935).

Mouse anti-CENP-A (Abcam-ab13939), rabbit anti-CENP-T and rabbit anti-Mis12 (donated from Ian Cheeseman), rabbit anti-CENP-B (donated from Don Cleveland's lab), rabbit anti-CENP-C (donated from Aron Straight's lab) and rabbit anti-MAD1 (donated from Mary Dasso's lab) were used at 1: 500 dilutions. Mouse-anti GFP (Abcam-ab290) was used at 1:1000 dilutions. Human ANA-centromere CREST autoantibody (Antibodies incorporated-15-234) was used at 1: 2000 dilutions. The secondary antibodies, goat anti-rabbit DY 488, goat anti-rabbit DY 594, goat anti-mouse DY 488, goat anti-mouse DY 594 and goat anti-human DY 647 (all from Thermofisher scientific) were used at 1: 500 dilutions.

For immunoblotting, mouse anti-CENP-A was used at 1: 300 dilutions. Rabbit anti-DAXX (Cell Signaling-25C12), and mouse anti-Tubulin were used at 1: 1000 dilutions. Rabbit anti-GAPDH (Thermofisher-MA5-15738) was used at 1: 2000 dilutions. HRP secondary antibodies against rabbit (Rockland-611-1322) and mouse (Rockland-610-1319) were used at 1:40,000 dilutions. Blots were treated with uniglow reagent (Rockland-Uniglow-0100) prior to film development.

### Microscopy and image analysis

Immunostained cells were imaged on Delta Vision Core system (Applied Precision / GE Healthcare, Issaquah, WA) consisting of Olympus IX70 inverted microscope (Olympus America, Inc. Melville, NY) with 100X NA 1.4 oil immersion objective and a CoolSnap HQ 12-bit camera (Photometrics, Tucson, AZ) controlled by SoftWoRX software. Filters used for imaging were FITC (Ex490/20; Em 528/38), RD-TR (Ex555/28; Em 617/73) and DAPI (Ex360/40; Em 457/50) of the 86000 Sedat Quadruple Filter Set (Chroma Technology Corp, Bellows Falls, VT). Z-stacks of at least 10 focal planes were acquired with an exposure of 0.1 to 0.5 seconds, depending on the filter. Signal intensity was measured using the plot profile tool in *Softworx*.

For live-cell imaging, cells were counted in a hemocytometer and seeded in LabTek 0.15 mm thickness glass dishes (Nunc). 24 hours after seeding, cells were synchronized using 1 μg/ml of Aphidicolin for 24 hours to arrest the cells in S-phase, followed by release into Aphidicolin-free media for 8 hours before imaging [66]. For nocodazole assay, cells were treated with 100 ng of Nocodazole before imaging. Media was changed into CO_2_ independent Leibovitz's L15 medium (Thermofisher Scientific-21083027) before imaging. Cells were maintained at 37°C in environmental chamber and imaged on Applied Precision Delta Vision Elite microscope (Applied Precision / GE Healthcare, Issaquah, WA) consisting of Olympus IX71 inverted microscope equipped with LED detectors and sCMOS pco. edge camera (PCO AG, Kelheim, Germany). For long-term live-cell imaging, images were acquired using a 40× NA 0.6 air objective (Olympus America, Inc. Melville, NY) as z-stacks with at least 3 focal planes with a step size of 3 μm, with 5 min time lapse for 4 hours total. Exposure was set to 0.1 seconds for FITC channel (Ex 475/28, Em 525/48) and for 0.1 sec for DIC channel. Timing of anaphase onset from NEBD was determined using GFP signal DIC images.

To prepare the figures, images were deconvolved with *Softworx* and scaled manually to 8-bit using linear LUT and the same range of scaling for all the images.

### Quantitative immunofluorescence analysis

To calculate fluorescence intensities, boxes of 8 × 8 pixels were drawn on centromeric region as ascertained by bright foci of CENP-A and/or CREST and on non-centromeric region as ascertained by the signal outside the centromeric region on a chromosome (chromosome spreads) or chromosomes aligned on the metaphase plate. For background, four boxes of 8 × 8 pixels were drawn at four random areas within the cytoplasm in the same cell. The maximum intensity values from all drawn areas were obtained using data inspector tool in *Softworx*. Final fluorescence intensity for each protein was calculated by subtracting the average background intensity. Intensity measurements were done for at least 10 centromeric and non-centromeric spots in each cell for an average of 10 cells from two independent experiments. For statistical analysis, average values from more than 100 centromeric or non-centromeric spots were calculated and used as a mean to calculate SEM across areas measured.

### Interkinetochore distance measurement

For interkinetochore distance we used the distance measurement tool in *Softworx* to draw a straight line between the brightest pixels of CENP-A or Nuf2 on two sister chromatids. Only congressed pairs of kinetochores in MG132 arrested metaphase cells were included for analysis. Orientation between two centromeric/kinetochore markers and focal plane were used as a basis for considering two kinetochores as a pair. For example, to consider two sister kinetochores as a pair in a cell immunostained with Nuf2 (outer kinetochore marker) and CENP-A (inner kinetochore marker), sister kinetochores should reside in the same focal plane and should orient Nuf2 towards the spindle pole and CENP-A towards the equatorial plate. The length of each line was then calibrated based on a units/pixel and assigned in μm. Interkinetochore distance was measured for at least 10 kinetochore pairs in a single cell and 8-15 cells from two independent experiments. Average values from more than 100 kinetochore pairs were calculated and used as the mean to calculate the SEM across areas measured.

### Statistical analysis

*P*-values were obtained using the Mann-Whitney U test or proportion test in *Prism* and R-lab, respectively.

## SUPPLEMENTARY MATERIALS FIGURES AND TABLES



## Abbreviations

CIN: Chromosomal instability
RPE1: Retinal Pigmental Epithelial
SAC: Spindle Assembly Checkpoint
CCAN: Constitutive Centromere Associated Network
MAD1: Mitotic Arrest Deficient
KMN: Knl1 Mis12 Ndc80
GFP: Green Fluorescent Protein
NEBD: Nuclear Envelope Breakdown
PFA: Paraformaldehyde
ANA: Anti-Nuclear Antibody
PBST: Phosphate Buffered Saline Tween
DAPI: 4’,6-diamidino-2-phenylindole
