# Supplementary material for: Mislocalization of centromeric histone H3 variant CENP-A contributes to chromosomal instability (CIN) in human cells
Source: Oncotarget. 2017 May 23;8(29):46781–800. doi: 10.18632/oncotarget.18108 (PMC5564523; doi:10.18632/oncotarget.18108)
Supplement: Supplementary file 1 [file oncotarget-08-46781-s001.pdf]

Mislocalization of centromeric histone H3 variant CENP-A contributes to chromosomal instability (CIN) in human cells

Supplementary Materials

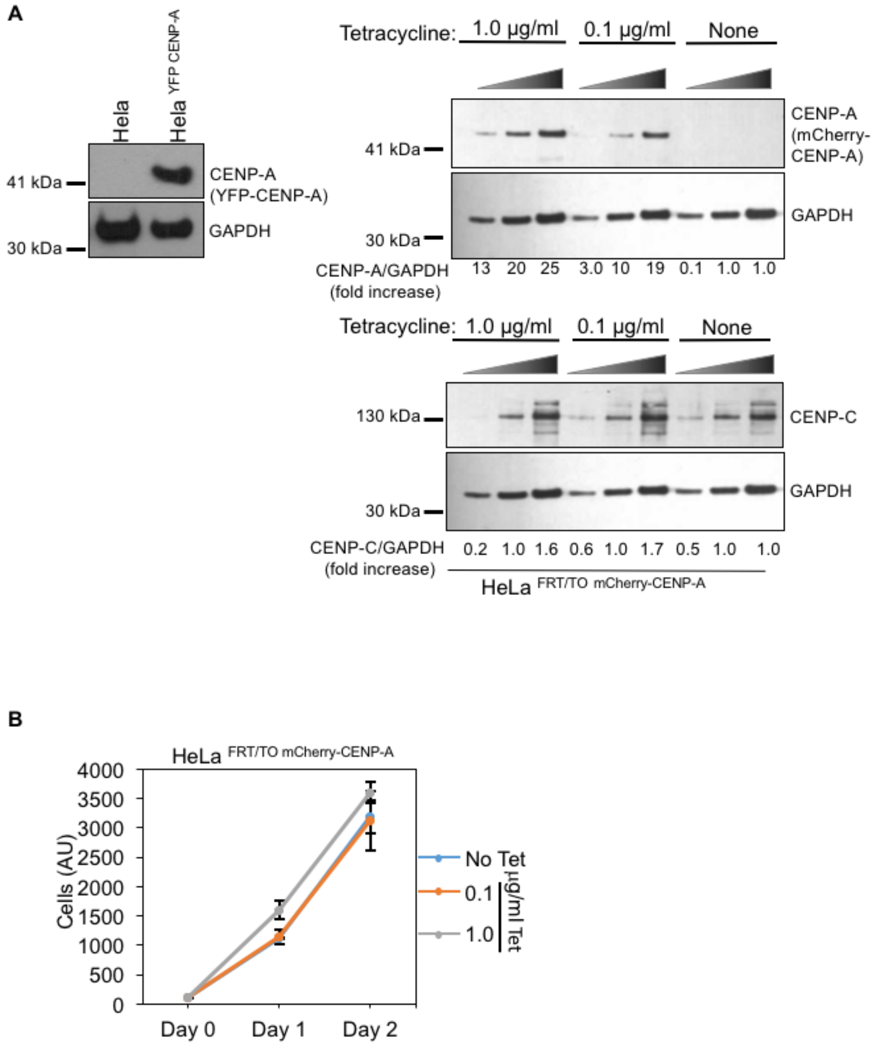

Supplementary Figure 1: Treatments with increased concentrations of tetracycline increase CENP-A levels without effecting the cell viability. (**A**) CENP-A expression increases with increased concentrations of tetracycline. Representative immunoblots show expression levels of YFP-CENP-A (left panel), mcherry-CENP-A (right, upper panel), and CENP-C (right, lower panel) in HeLa $^{\text{YFP-CENP-A}}$ cell lysates and HeLa $^{\text{FRT/TO cherry-CENP-A}}$ cell lysates treated with different concentrations of tetracycline. Cell lysates were collected with 2x Laemmle buffer and loaded in serial dilutions for western blot analysis (right panels). Blots were then probed with antibody against CENP-A to assess CENP-A levels. GAPDH was used as a loading control. (**B**) CENP-A overexpressing cells are viable. Graph shows proportion of viable HeLa $^{\text{FRT/TO cherry-CENP-A}}$ cells treated with indicated concentrations of tetracycline. Y-axis corresponds to proportion of viable cell in arbitrary units. After treating cells with tetracycline for days indicated, cells were treated with 0.8% Trypan blue (final concentration 0.4%) for five minutes. Cells were then counted in a Neuberger chamber for live (colorless) and dead (blue colored) cells from which the proportion of viable cells was then derived. Error bar represents standard error of mean (SEM) across three independent experiments. Day 0 represents the day when cells were not treated with tetracycline, Day 1 represents 24 hours post treatment with tetracycline and Day 2 represents 48 hours post treatment with tetracycline.

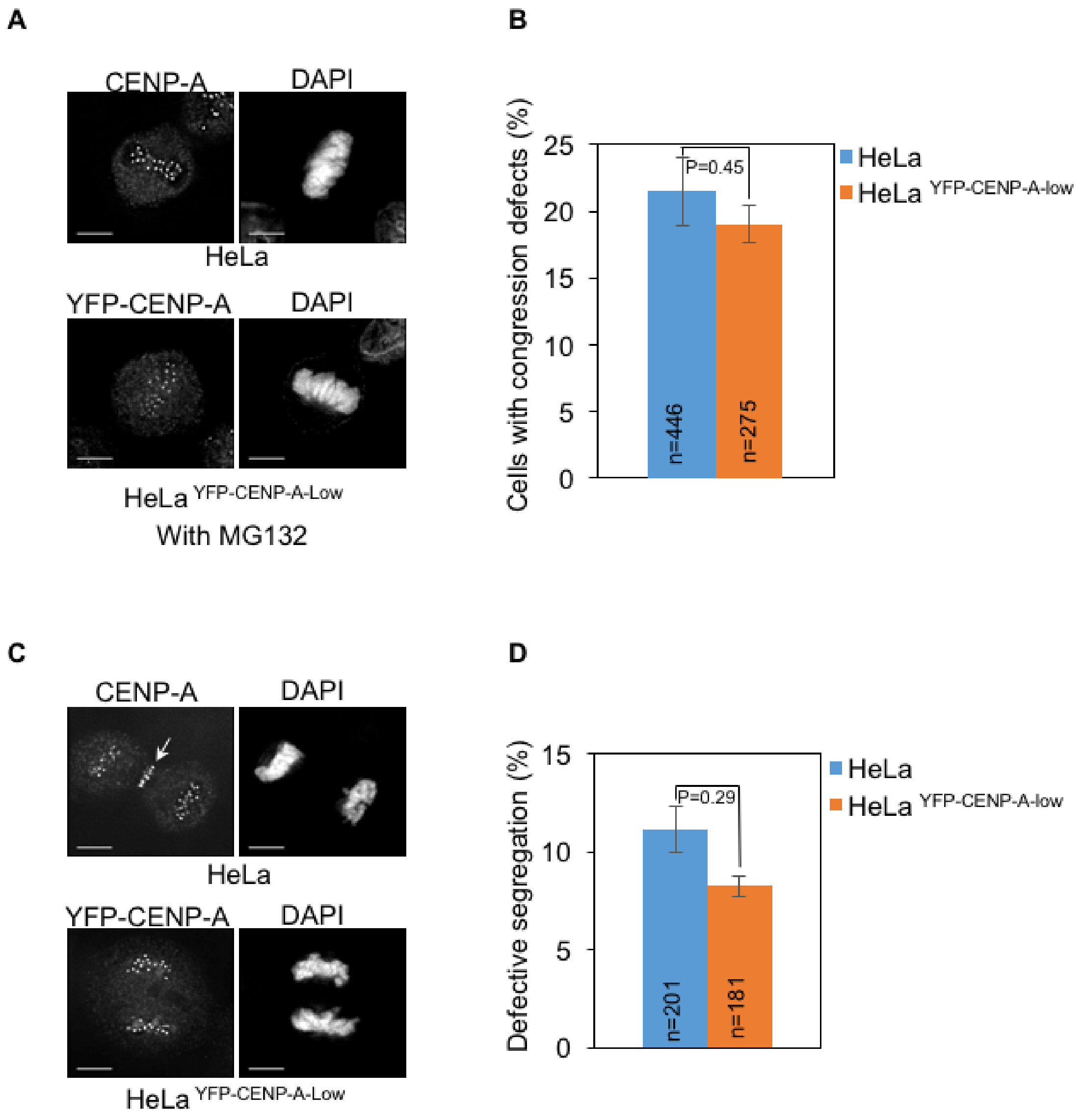

Supplementary Figure 2: Overexpressing low amount of YFP-CENP-A does not cause mitotic defects in human cells. (**A-B**) Overexpression of low amount of YFP-CENP-A does not contribute to chromosome congression defects. Representative immunofluorescence images of HeLa (upper panel) and Hela $^{\text{YFP-CENP-A}}$ cells expressing lower amount of CENP-A (Hela $^{\text{YFP-CENP-A-low}}$) (lower panel) show chromosome congression status. Prior to immunostaining, cells were treated with 10 µM MG132 for three hours. Cells were immunostained with antibodies against CENP-A for HeLa and GFP for Hela $^{\text{YFP-CENP-A-low}}$ to visualize CENP-A. Scale bar: 5 µm. (B) Proportion of cells with defective chromosome congression is comparable between HeLa and HeLa $^{\text{YFP-CENP-A-low}}$ cells. Bar chart shows proportion of HeLa and Hela $^{\text{YFP-CENP-A-low}}$ cells with defective chromosome congression. (**C-D**) Overexpression of low amount of YFP-CENP-A does not contribute to chromosome segregation defects. Representative immunofluorescence images of HeLa (upper panel) and Hela $^{\text{YFP-CENP-A-low}}$ (lower panel) show chromosome segregation status. Cells were immunostained with antibodies against CENP-A for HeLa and GFP for Hela $^{\text{YFP-CENP-A-low}}$ to visualize CENP-A. Scale bar: 5 µm. (D) Proportion of cells with chromosome segregation defects is comparable between HeLa and HeLa $^{\text{YFP-CENP-A-low}}$ cells. Bar chart shows proportion of HeLa and Hela $^{\text{YFP-CENP-A-low}}$ cells with chromosome segregation defects. Error bar represents standard error of mean (SEM) across three independent experiments. 'n' denotes number of cells analyzed. P values calculated using proportion test are indicated.

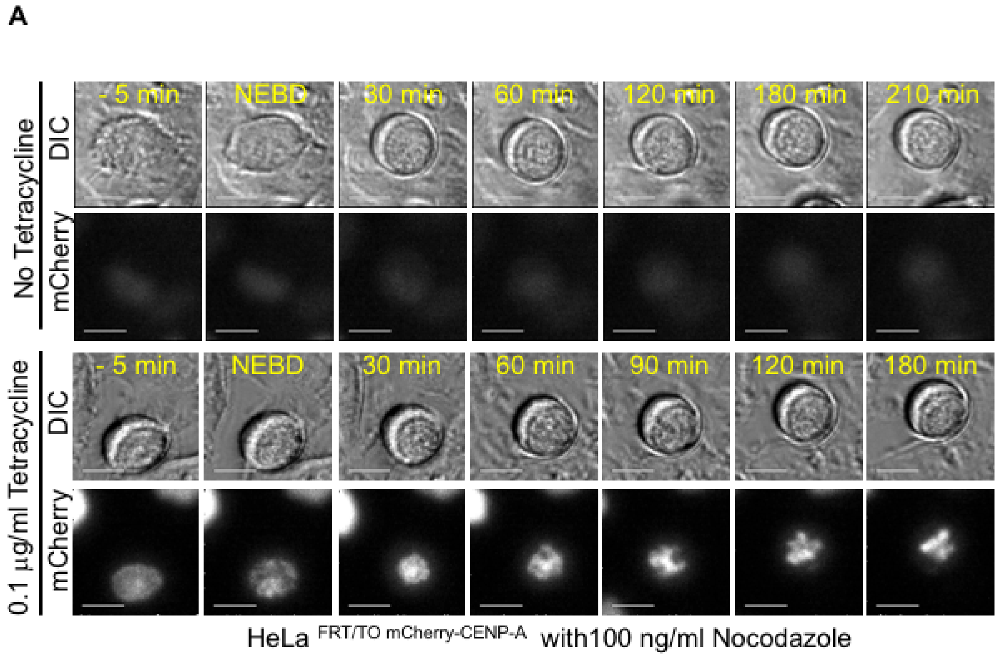

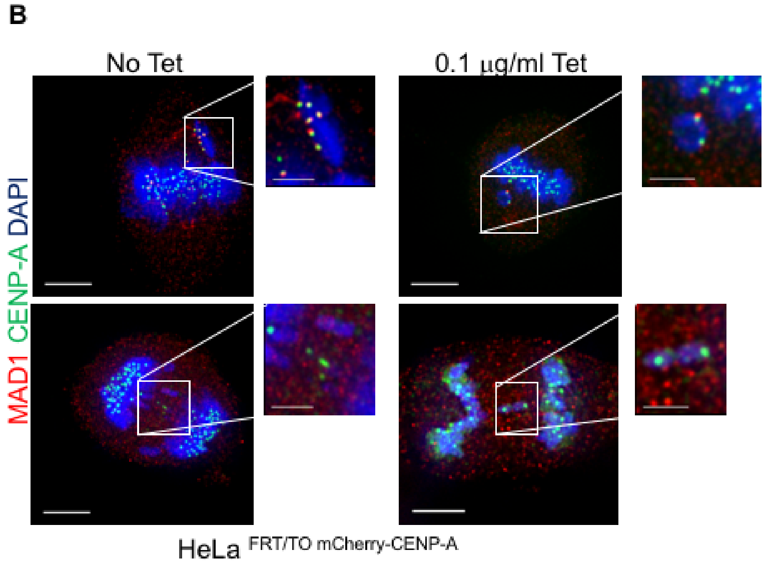

Supplementary Figure S3: Spindle assembly checkpoint is intact in CENP-A overexpressing cells. (A) Nocodazole-treated CENP-A overexpressing cells arrest in mitosis. Still images from time- lapse analysis of HeLa $^{FRT/TO\ mcherry\text{-}CENP\text{-}A}$ cells treated or untreated with tetracycline. Prior to imaging, cells were treated with 100 ng/ml nocodazole. Cells were then imaged every five minutes for eight hours, but still images are shown from indicated time points only. CENP-A expression was ascertained by mCherry signal in tetracycline-treated cells and mitotic acceleration was ascertained using DIC panels. Scale bar: 25 µm. (B) MAD1 is present in CENP-A uncongressed chromosomes with CENP-A overexpression. Representative immunofluorescence images of HeLa $^{FRT/TO\ mCherry\text{-}CENP\text{-}A}$ cells treated or untreated with 1.0 µg/ml tetracycline showing MAD1 expression. Cells were immunostained with antibodies against CENP-A and MAD1 and stained with DAPI for DNA. Insets correspond to boxed areas in main images. Scale bar: 5 µm for main images and 2 µm for insets.

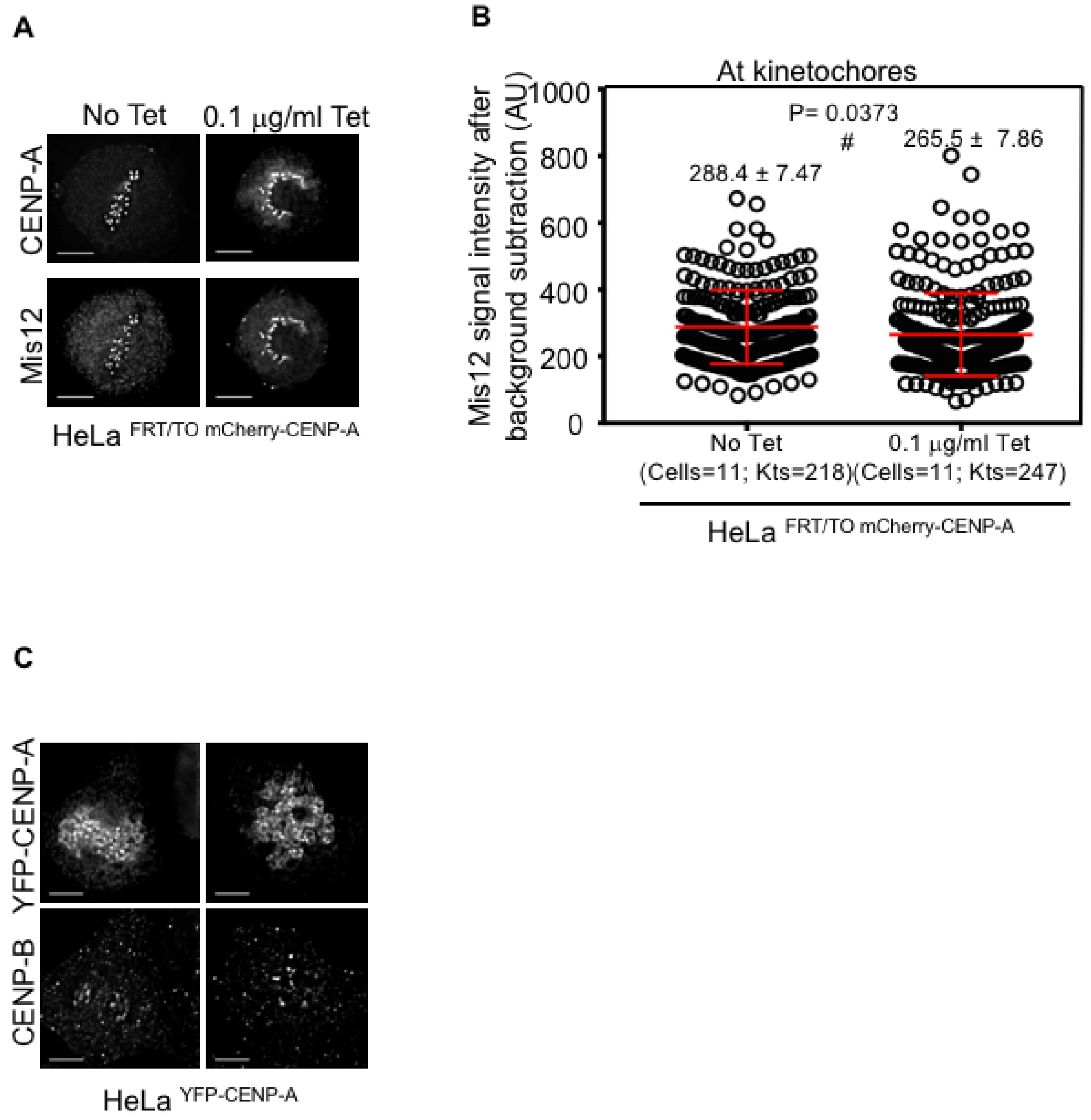

Supplementary Figure S4: Mis12 and CENP-B are not altered in CENP-A overexpressing cells. (**A**) Mis12 does not mislocalize in CENP-A overexpressing cells. Representative immunofluorescence images of HeLa $^{\text{FRT/TO mCherry-CENP-A}}$ cells untreated and treated with 0.1 µg/ml tetracycline show normal localization of Mis12. Cells were immunostained with antibodies against Mis12, CENP-A, and CREST antisera for centromeres. Scale bar: 5 µm. (**B**) Mis12 levels were not altered at kinetochores in CENP-A overexpressing cells. Prism graph shows Mis12 signal intensity at kinetochore in metaphase plate of HeLa $^{\text{FRT/TO mCherry-CENP-A}}$ cells untreated and treated with 0.1 µg/ml tetracycline. Each circle represents a kinetochore and ‘Kts’ denotes number of kinetochores analyzed in certain number of cells as denoted by ‘cells’. Red horizontal lines represent mean signal intensities as indicated. Error bars represent standard error of mean (SEM) across the kinetochores measured from three independent experiments. *P*-value calculated using the Mann-Whitney U test is indicated. # represents statistically insignificant values. (**C**) CENP-B does not mislocalize in CENP-A overexpressing cells. Representative immunofluorescence images of HeLa $^{\text{YFP-CENP-A}}$ cells show normal localization of CENP-B. Cells were immunostained with antibodies against CENP-B and GFP for CENP-A and CREST antisera for centromeres. Scale bar: 5 µm.

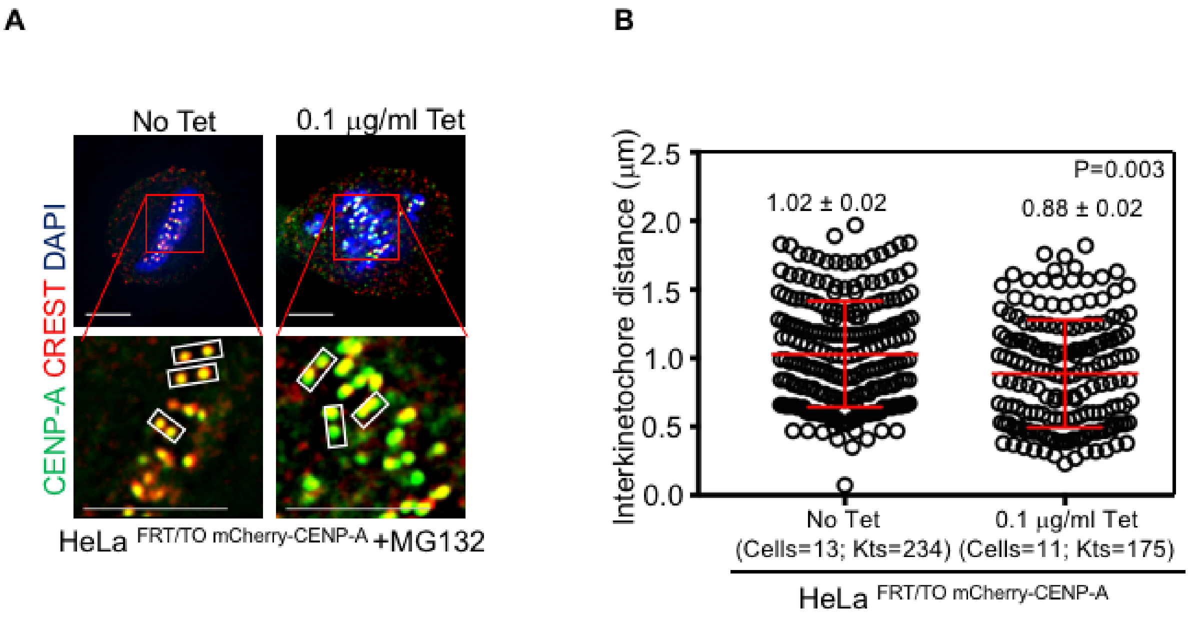

Supplementary Figure S5: Interkinetochore distance is reduced in CENP-A overexpressing cells, resulting in weaker kinetochore-microtubule attachments. (**A**) Interkinetochore distance is reduced in CENP-A overexpressing cells. Representative immunostained images of HeLa $^{FRT/TO\ mCherry\text{-}CENP\text{-}A}$ cells untreated and treated with 0.1 µg/ml tetracycline show interkinetochore distance. Prior to immunostaining, cells were treated with 10 µM MG132 for three hours. Cells were then immunostained with antibodies against CENP-A and CREST antisera for centromeres and stained with DAPI for DNA. Insets correspond to red boxed areas in main images. White boxed areas in the insets show kinetochore pairs included in the analysis. Scale bar: 5 µm. (**B**) Prism graphs show interkinetochore distance as ascertained by distance between two CENP-A signals within aligned chromosomes in MG132-arrested HeLa $^{FRT/TO\ mCherry\text{-}CENP\text{-}A}$ cells untreated and treated with 0.1 µg/ml tetracycline. Each circle represents a pair of kinetochores and 'Kts' denotes number of kinetochore pairs analyzed in certain number of cells as denoted by 'cells'. Red horizontal lines represent the mean interkinetochore distance as indicated. Error bars represent standard error of mean (SEM) across pairs of kinetochores analyzed from two independent experiments. *P*-values calculated using the Mann-Whitney U test are indicated.
